# First Record of *Triops cancriformis* (Branchiopoda: Notostraca) in Qatar: Its Origin and Range Expansion Inferred From Genetics, Morphology, and Reproduction

**DOI:** 10.1002/ece3.72673

**Published:** 2025-12-15

**Authors:** Kuei‐Chiu Chen, Ramaswamy Narayanaswamy

**Affiliations:** ^1^ Division of Premedical Education Weill Cornell Medicine ‐ Qatar, Education City, Qatar Foundation Doha Qatar; ^2^ College of Health and Life Sciences Hamad Bin Khalifa University, Education City, Qatar Foundation Doha Qatar

**Keywords:** 16S, Arabia, COI, dispersal, Middle East, rain pool, tadpole shrimp

## Abstract

The presence of *Triops cancriformis* has not been broadly reported outside Europe or North Africa. Among the few exceptions, including the studies on the Middle Eastern populations, genetic information is almost completely absent. This study confirms the presence of *T. cancriformis* as a single population in Rawdat Nu'man, Qatar, using both morphological and genetic data. Their carina and telson were consistent with the traits in the subspecies of *T. c. simplex,* while the COI and 16S sequence analysis revealed that the population of Rawdat Nu'man is consistent with *T. c. cancriformis.* As this morphology vs. genetics inconsistency has been reported previously, the observation in the Qatari *T. cancriformis* further calls for reassessment of the status of the two subspecies. The COI‐based phylogeny shows the Nu'man population being most closely related to a population in Königswartha, Germany. Further, the presence of only female individuals, the absence of genetic diversity, and limited local distribution indicate recent colonization in Qatar, possibly through passive dispersal via diapausing cysts carried by avian vectors during seasonal migrations.

## Graphical abstract

First record of *Triops cancriformis* is reported in Qatar using genetic, morphological, and reproductive data. The closest relative of this population is found in Germany. Dispersal of propagules is believed to be via avian seasonal migration.

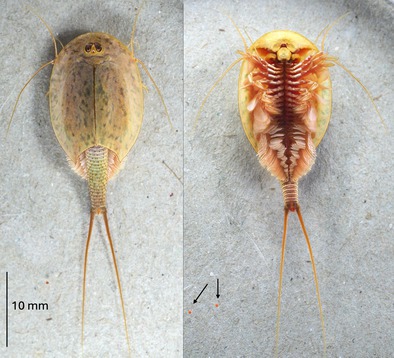

## 1 Introduction

Branchiopoda consists of species that have adapted to a life cycle made up of a relatively short and fast developmental period in temporary rain pools and drought‐tolerant cysts that can withstand decade‐long desiccation, hatching only when the seasonal pools return (Longhurst 1955a; Cesari et al. 2004; Atashbar et al. 2014). With the small and drought‐tolerant cysts, they have a strong ability to disperse and settle in even the most outlying locations (Longhurst 1955a). In areas that are arid or semi‐arid, members of the order Notostraca frequently exist as the largest branchiopods and have a close to global distribution (Brendonck et al. 2008; Vanschoenwinkel et al. 2012). There is only one family, Triopsidae, classified under Notostraca, and it comprises only two genera, both of which contain an unresolved number of species. In *Lepidurus*, up to around nine species are reported, while there may be between 20 and more than 50 phylogenetic species in *Triops* when including those found in Australia and Africa (King and Hanner 1998; Korn et al. 2013; Mathers et al. 2013; Korn and Hundsdoerfer 2016; Meusel and Schwentner 2017; Rogers et al. 2021).

Notostraca is made up of species that may be gonochoric, hermaphroditic, or androdioecious, with non‐gonochorism evolving multiple times independently in the lineages from the gonochoric ancestors. This variable trait is in contrast with their morphological stasis in the 250 million years of evolution (Mathers et al. 2013). It has been hypothesized that multiple colonization events from the androdioecious and hermaphroditic members may have occurred in high latitudes during post‐glacial range expansions in some of the species (Mathers et al. 2013).

In the genus *Triops*, *T. cancriformis* (Bosc, 1801) is often referred to as the European *Triops*, which indeed has a distribution primarily in Europe, the UK, and islands in the Mediterranean (e.g., Zierold et al. 2007). Their presence, however, has been reported in northern Africa, the Middle East, and Asia, including the Arabian Peninsula, Indian subcontinent, and Japan, where their habitats may be temporary rain pools as well as cultivated rice fields (Thiéry 1996; Suno‐uchi et al. 1997; Golzari et al. 2009; Padhye and Ghate 2016). The reproductive system of the species is particularly labile among all other species in the order, showing all three reproductive modes in different populations (Mathers et al. 2013). Among them, the gonochoric populations are restricted to the Iberian Peninsula and northwestern Africa, while the hermaphroditic and androdioecious populations are found elsewhere in Eurasia (e.g., Zierold et al. 2007; Padhye and Ghate 2016).

Among the *Triops* species, *T. granarius* perhaps shows the widest global distribution and exhibits a completely gonochoric reproductive mode in all its populations (e.g., Longhurst 1955b; Korn and Hundsdoerfer 2006). In Qatar, a previous study using mitochondrial and nuclear markers has reported a widespread presence of *Triops c.f. granarius* in seasonal rain pools (Chen et al. 2023). In this study, we report the findings of *T. cancriformis* that share its habitat with *T. granarius* in the largest rain pool of Rawdat Nu'man in northern Qatar. Both morphological traits and genetic traits from COI and 16S sequences were used to confirm the species. The reproductive mode of the population is further examined to compare with other populations reported in the Middle East (e.g., in Iran in Golzari et al. 2009, and in Saudi Arabia, as in Hassan 2015). Phylogenetic analysis is conducted to determine the pattern of lineages and to identify the possible origin of the Nu'man population using sequences available from the GenBank of the US National Center for Biotechnology Information (NCBI). The possible mechanism of dispersal from the source population is also discussed in this report.

## 2 Methods

### 2.1 Field Site Description

**FIGURE 1:**
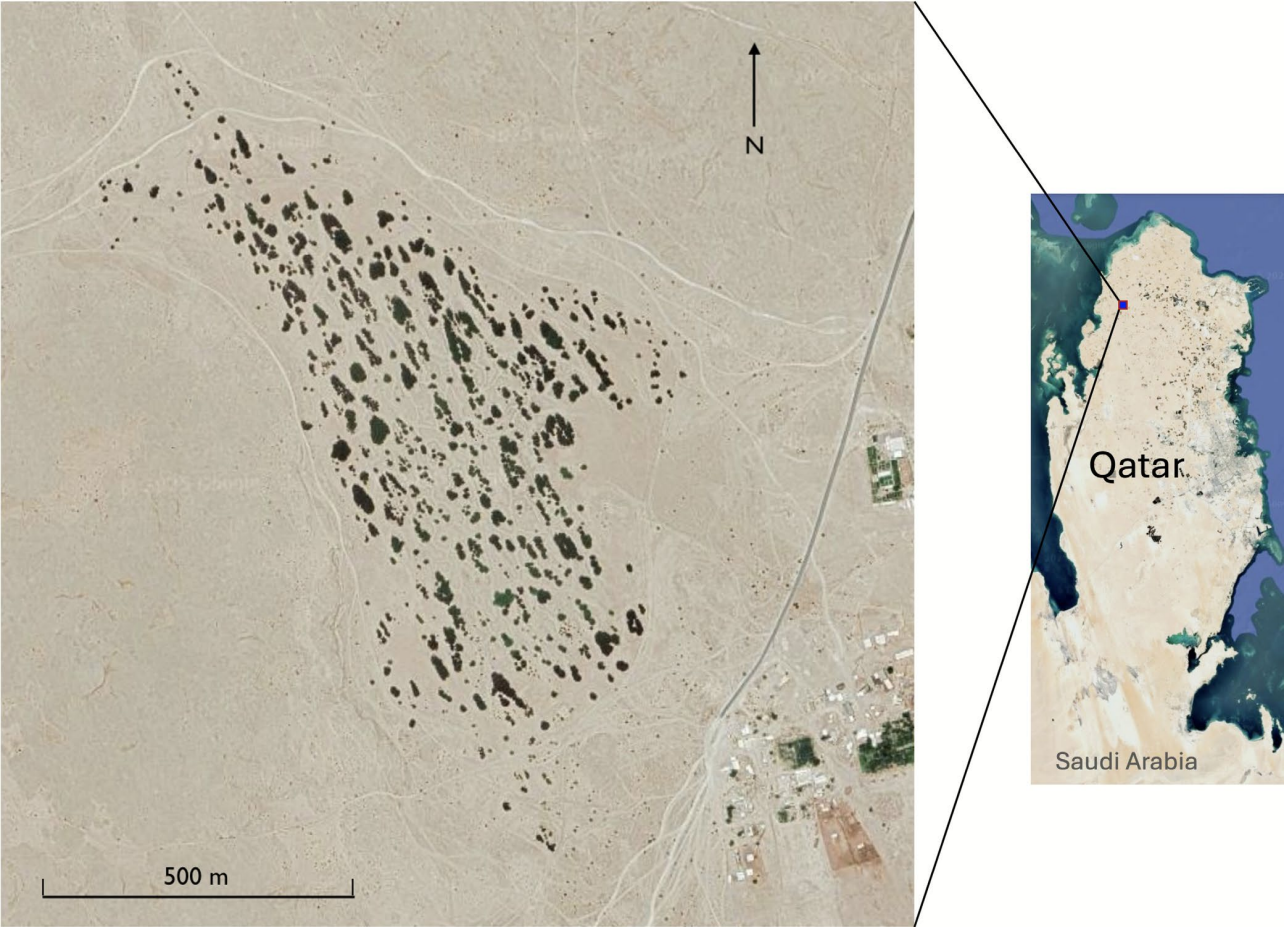
Rawdat Nu'man (lat 25°51′42.7′′ N long 51°04′36.6′′E) in northern Qatar. The aerial view shows the rawdat, the Arabic word of fertile depressions with green vegetation, that is covered in the dark green patches of sidra trees (*Ziziphus spina‐christi*) predominantly. Part of the small village of Nu'man is seen at the right of the aerial photo across the country road with pavement ending in front of the village. (Images modified from Google Maps.)

**FIGURE 2:**
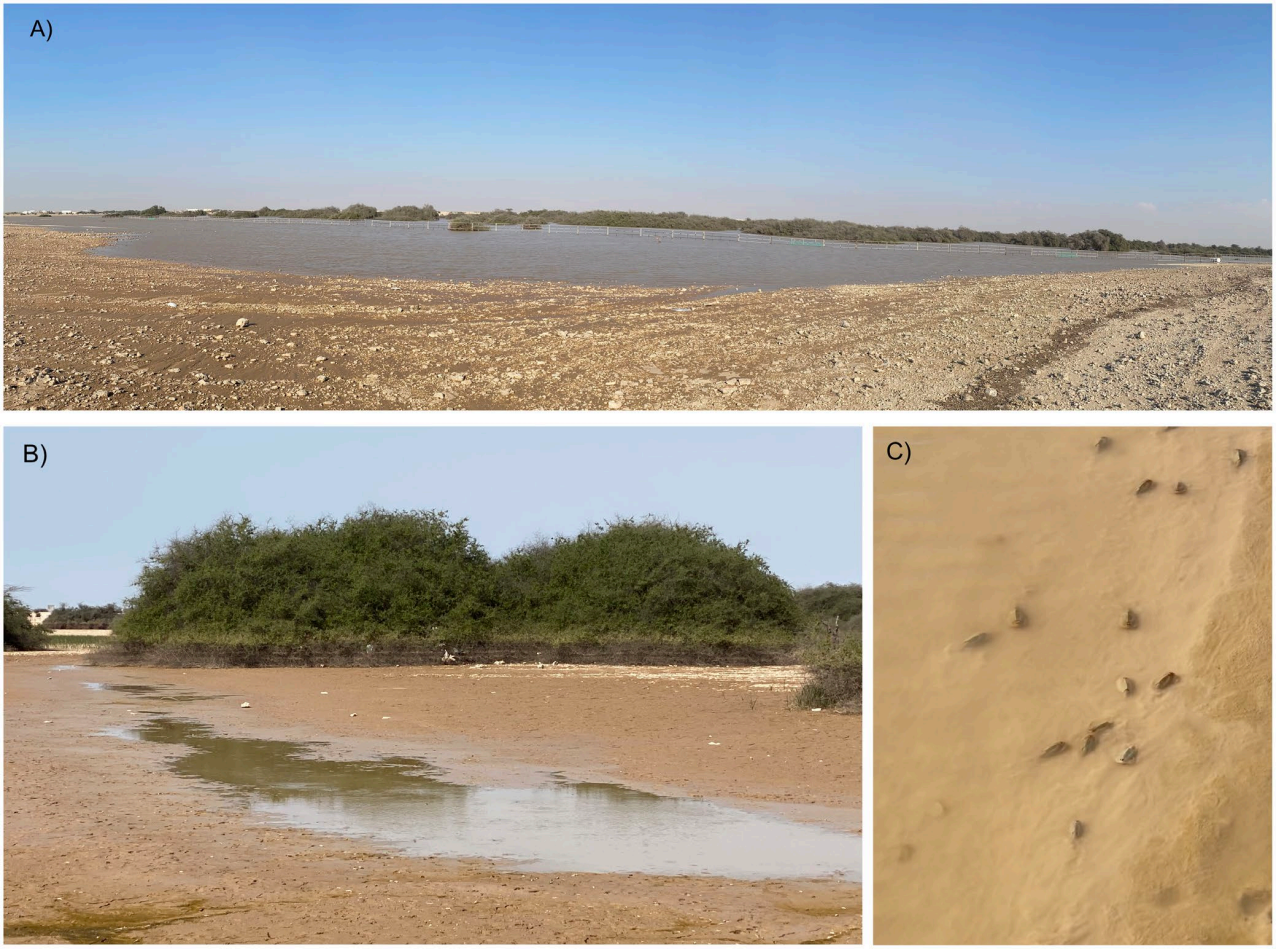
(A) East‐facing, partial view of the flooded Rawdat Nu'man on January 7, 2022. The floods far exceeded the protected white fences surrounding the perimeter. In this image, the entire span between the left and the right is less than 200 m. (B) After six weeks of evaporation, isolated shallow pools or channels remained. The water line is seen near the base of *Ziziphus spina‐christi* in the far ground. (C) *Triops* individuals in the shallow water.

Rawdat Nu'man (lat 25°51′42.7′′ N long 51°04′36.6′′E, Figure 1) is located in northern Qatar, where higher precipitation is often observed compared to the southern part of the country (*The Peninsula* 2022). In the rawdat, an Arabic term describing fertile depressions often with lush vegetation, sidra trees (*Ziziphus spina‐christi*) usually constitute the dominant and perennial flora. In Qatar, rawdat (rawda as the singular form) are distributed primarily in the northern half of the peninsula because of heavier precipitation. Since these areas have higher biodiversity compared to the rest of the terrestrial habitats in Qatar, many, if not most, of the rawdat have been listed as protected land (personal observation). Depending on the size of the rawdat, after a heavy rain storm, the base of the *Z. spina‐christi* trees is submerged for weeks. In Rawdat Nu'man, being among the largest in the country, the submersion may last a couple of months or longer, and the size of the rain pool may cover nearly 1 km^2^ and exceed the area covered by *Z. spina‐christi* and the protective fences (Figure 2A). Between December 31, 2021, and January 19, a few rain storms reached Qatar, with the cumulative precipitation of 17 mm in the northern part of the peninsula (Visual Crossing, at https://www.visualcrossing.com/weather‐history). The water in the rain pool persisted until at least February 18, 2022. In that year, since the pool was deep (might be > 80 cm at the deepest part as estimated) and the water murky in the first month after the rains, sample collection did not start until five weeks after the pool formation. The collections were eventually made on February 4th, 11th, and 18th when the water was shallow enough to wade in and the specimens were easier to observe (Figure 2B,C). In spring 2024, multiple rain storms occurred in Qatar between February 11 and April 16, with cumulative precipitation reaching 107 mm in Nu'man (https://www.visualcrossing.com/weather‐history), and the rain pool was maintained until the end of May. A collection was made only near the end of the pool's longevity, on May 19, 2024. In general, we used a small bowl instead of a dip net to scoop up the specimens when they emerged from the depths to minimize the damage caused by the struggle. In each of these visits, between 20 and 35 specimens were collected.

### 2.2 Morphological Study

**FIGURE 3:**
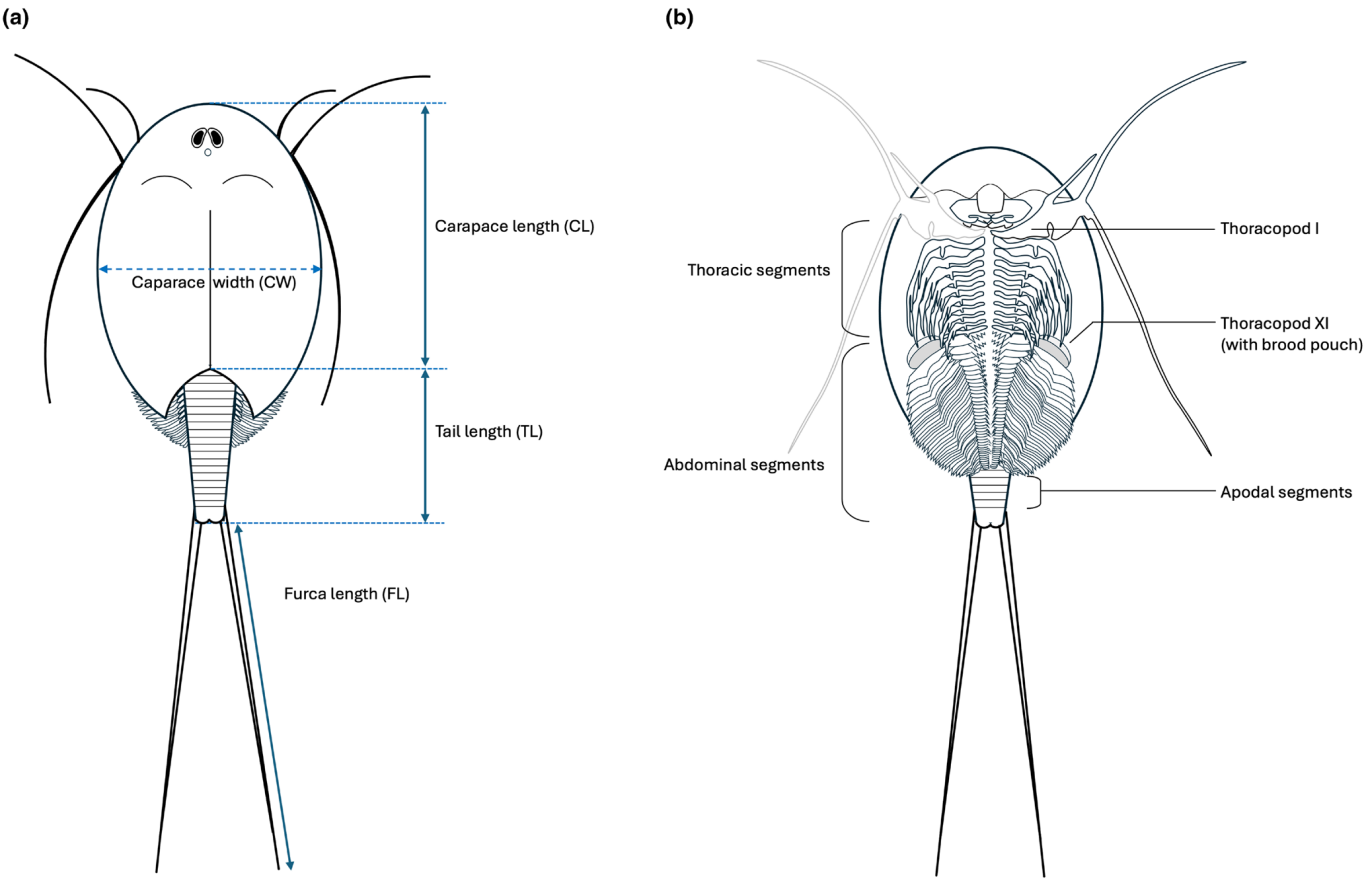
(A) Simplified sketch of the dorsal view of *Triops cancriformis simplex* and the measurements recorded in the study. (B) Schematic depiction of the ventral view and the structures measured of *T. cancriformis* .

Morphometry of each specimen was made by measuring the length, width, and depth of the carapace, as well as the length of the exposed abdomen and the furca (Figure 3). The length of the carapace was measured from the anterior terminus along the carina to reach the sulcus, the width as the widest span, and depth as the greatest thickness of the carapace. In addition, the carina was also subject to assessment for the presence of ridges or spikes. In this trait, spikes located on the apodal segments from both the ventral and dorsal sides were studied and compared to the diagram in Longhurst (1955b) to determine individual traits that may distinguish subspecies of *T. cancriformis* . The morphology of the sulcus and the presence or absence of spines on the telson were also noted. Additionally, the number of segments in the thorax and in the abdomen, including the apodal segments, were counted (Figure 3). The number of abdominal appendages was not counted because of the high variability of this trait (Longhurst 1955b). Because of possible contraction of the abdomen due to preservation (Longhurst 1955b), the number of exposed segments (not covered by the carapace) could not be reliably measured from these preserved specimens. Therefore, the proportion of exposed abdomen to the carapace was measured to the nearest mm using photographs of live specimens raised in the laboratory.

### 2.3 Tissue Digestion and DNA Sequencing

The right thoracic appendages numbers 2–4 were removed from 23 individuals (16 collected in 2022 and seven from 2024) for tissue digestion of up to 16 h followed by DNA extraction (DNeasy Blood & Tissue Kit, Qiagen). DNA concentrations were determined by NanoDrop 2000 Spectrophotometer (Thermo Scientific). PCR reactions were conducted in 25 μL volumes containing ~60 ng of template DNA and 1 μL each of 10 μM forward and reverse primers of CrustD (Radulovici et al. 2009) for the COI locus as the barcoding sequence (Hebert et al. 2003) and 16Sar and 16Sbr for the 16S rDNA (c.f. Murugan et al. 2002). *Taq* 2X Master Mix (New England BioLabs) at 12.5 μL was used on the ProFlex PCR System (Applied Biosystems) following the thermal programs mentioned in the respective publications above. Confirmation of the presence of amplicons was performed on 1.2% agarose gels with SYBR Gold Nucleic Acid Gel Stain (Thermo Fisher Scientific) and then purified by PureLink PCR Purification Kit (Thermo Fisher Scientific). The purified amplicons were quantified by Qubit Fluorometric Quantitation system (Thermo Fisher Scientific). Sanger sequencing was conducted on ABI3130X Genetic Analyzer (Applied Biosystems) with 5 to 20 ng of each sample of PCR amplicon. Sequencing in both the forward and reverse directions was performed, and the trace data were visualized and their quality verified using 4Peaks (Griekspoor and Guoothuis 2004). The forward and reverse complement sequences were aligned on CLC Main Workbench (v.25, Qiagen) to confirm the consistency of the sequence complementarity for each individual and to examine the sequence divergence among individuals within the population.

### 2.4 Phylogenetic and Haplotype Network Analysis

The forward and reverse sequences of each specimen were aligned to produce consensus sequences. In COI, only one single haplotype was produced, and the forward sequence of this haplotype was used to conduct a BLAST search on NCBI. The resulting sequences that showed at least 97% similarity were retained in the subsequent analysis. This generated 67 records in the databases, two of which were eliminated for being too short (less than 500 bp). The remaining 65 sequences, along with the only haplotype sequence in the local population, were aligned using CLC Main Workbench v. 25 (Qiagen Inc. 2025) to determine identical haplotypes collected from the same country. Only one copy of a unique haplotype from each country was included in the subsequent analysis in order not to over‐represent source countries with a larger sequence sample size deposited on GenBank. The sequences with unknown collection sites were also not included unless they represented unique haplotypes. This treatment then retained 47 sequences for the phylogenetic analysis on the COI sequences. Network analysis was conducted on the same group of sequences using PopART (Leigh and Bryant 2015) on the minimum span network setting. This setting ignores unresolved nucleotide bases, so consequently, the sample size was reduced to 25 sequences in the haplotype network analysis. In the 16S sequence analysis, again, all samples showed only one haplotype. Similarly, the forward sequence was used to conduct a search for similar sequences on GenBank where 39 sequences listed as *T. cancriformis*. Among these sequences, 28 were later retained for showing the country of origin of the corresponding specimens. Among the retained sequences, some were reverse sequences (AY159571‐AY159579), so they were processed to obtain their reverse complement sequences using CLC Main Workbench. The list of accession numbers of the retained COI and 16S sequences from the BLAST results is available in the Dryad data repository (see Data availability statement for link).

For the phylogenetic analysis using the COI marker, a *T. mauritanicus* sequence (Accession no. FN691442) was chosen as the outgroup. These sequences were aligned using Muscle on MEGA v. 11 (Tamura et al. 2021) and inferred using the maximum likelihood method with the nucleotide substitution model initially tested in raxmlGUI 2.0 (Edler et al. 2020). The analysis was then conducted using the HKY model and Gamma distribution (median setting) determined by the model test and under thorough bootstrap + consensus with 10 runs, each with 1000 replications. FigTree 1.44 (Rambaut and Drummond 2012) was used to visualize the final consensus tree.

## 3 Results

### 3.1 Morphological Analysis

**FIGURE 4:**
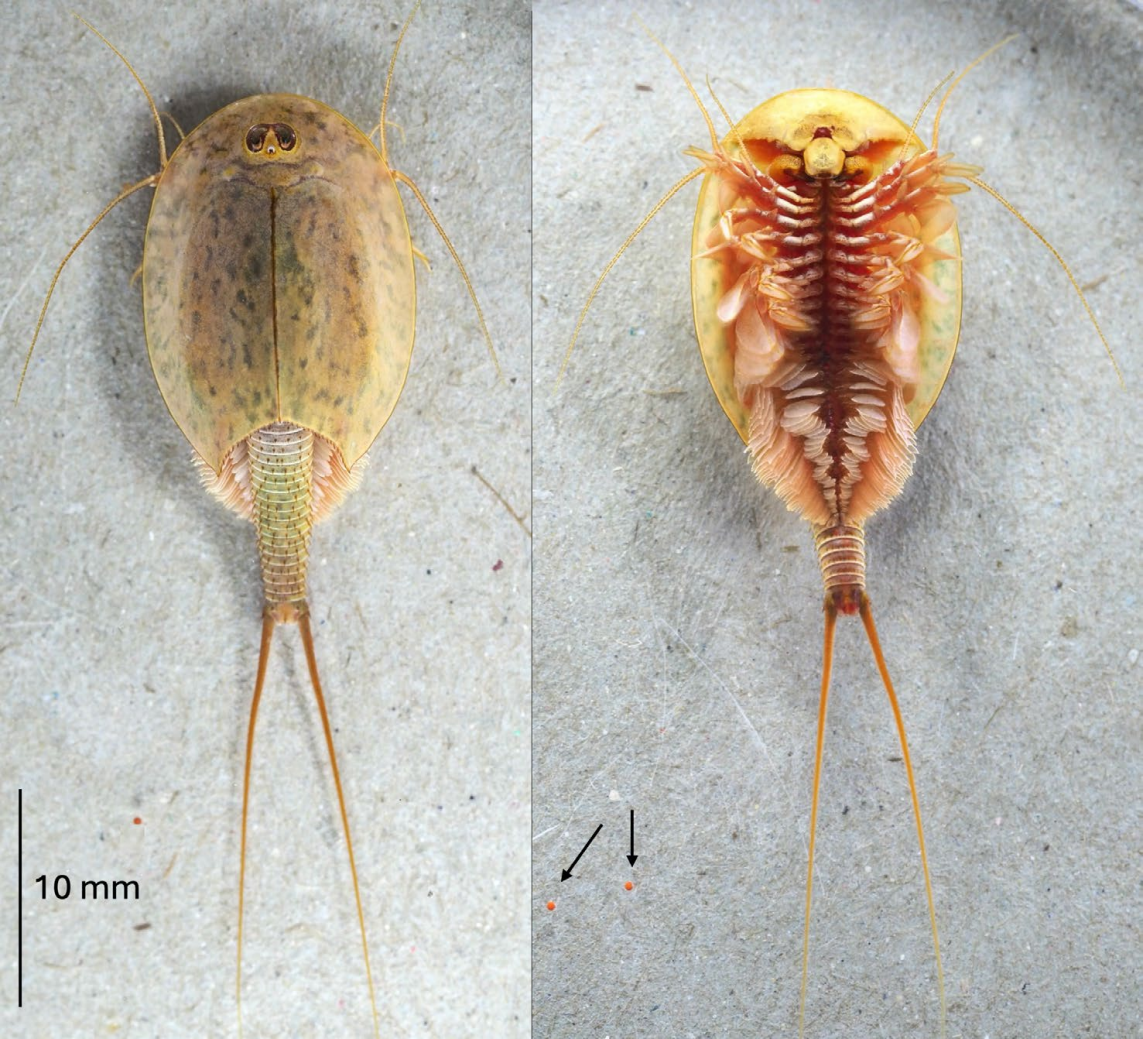
Gross morphology of a ~60‐day‐old *Triops cancriformis* from Rawdat Nu'man, Qatar, in natural coloration, left, dorsal view and right, ventral view. Two eggs are indicated by the arrows in the right image.

**FIGURE 5:**
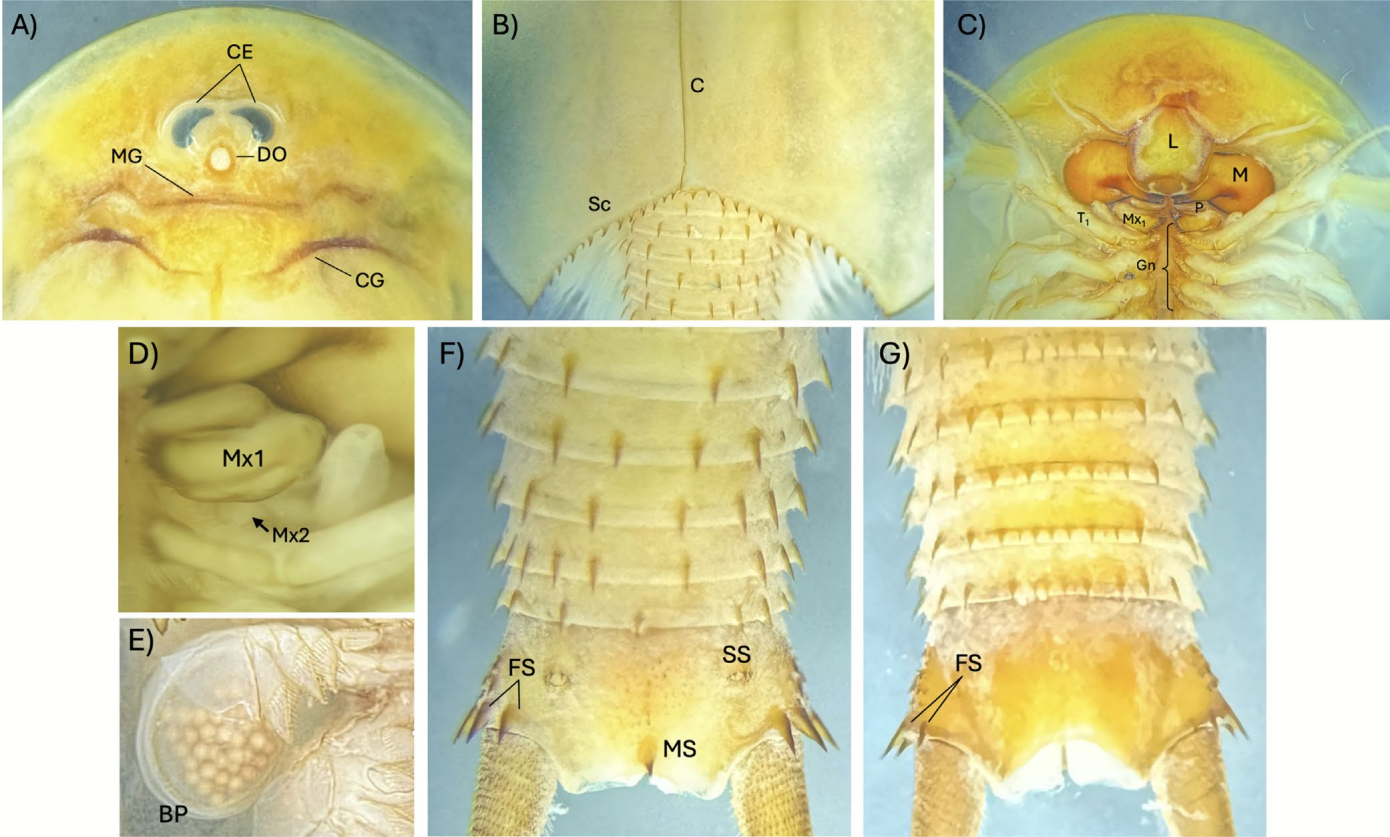
(A–G) Close‐up views of a preserved mature specimen. (A) Anterior dorsal view: CE, compound eyes, DO, dorsal organ, MG, mandibular groove, CG, cervical groove; (B) C, carina, Sc, sulcus, (C) anterior ventral view: L, labrum, M, mandible, P, paragnath, Mx_1_, first maxilla, T_1_, first thoracic appendage, Gn, gnathobase; (D) Mx2, second maxilla indicated by the arrow; (E) brood pouch (BP) showing > 30 eggs; (F) dorsal view of apodal segments, FS, furcal spine, MS, medial spine, SS, setal spine. (G) ventral view of apodal segments.

The gross morphology of a live specimen shows an oval shield‐shaped carapace in a medium brown or sandy base color covered with small, dull green irregularly shaped speckles (Figure 4). The close‐up images show the features of a typical *Triops cancriformis* (Longhurst 1955b): a round dorsal organ, a round arch of the sulcus, carina smooth without any spines (Figure 5A–C); all individuals showed their second maxillae (Figure 5D) and a pair of brood pouches at the 11th thoracic appendage (Figure 5E). On the dorsal side of the telson, a prominent medial spine was shown (Figure 5F). The apodal segments exhibited pointed spines in each segment on the dorsal surface (Figure 5F). These spines are somewhat smaller on the ventral side, and the supernumeraries were absent (Figure 5G). Detailed morphometry was conducted on 106 specimens collected in 2022 and 2024. Similar to the description of female *T. cancriformis* (Longhurst 1955b), the number of exposed segments was around 19, and 14 of the segments were covered by the carapace in the live specimens (Figure 4, further details below). These constituted a total of 33 segments in a great majority of the specimens. Also, the numbers of segments in each of the body parts were quite uniform (Table 1), with all individuals showing 11 thoracic segments. For the abdomen, a great majority of the specimens exhibited 22 segments, of which five were apodal (Figure 5E,F). Only four specimens (< 4%) had 23 abdominal segments, and one individual showed an incomplete posterior‐most segment bordering the telson.

**TABLE 1:** Number of segments in each body part on specimens collected in 2022 and 2024.

Body part	Number of segments (sample size)
Thoracic segment	11 (*n* = 106)
Abdominal segment	22 (*n* = 101), 23 (*n* = 4), 21.5 (*n* = 1)
Apodal segment (of abdomen)	5 (*n* = 106)

**FIGURE 6:**
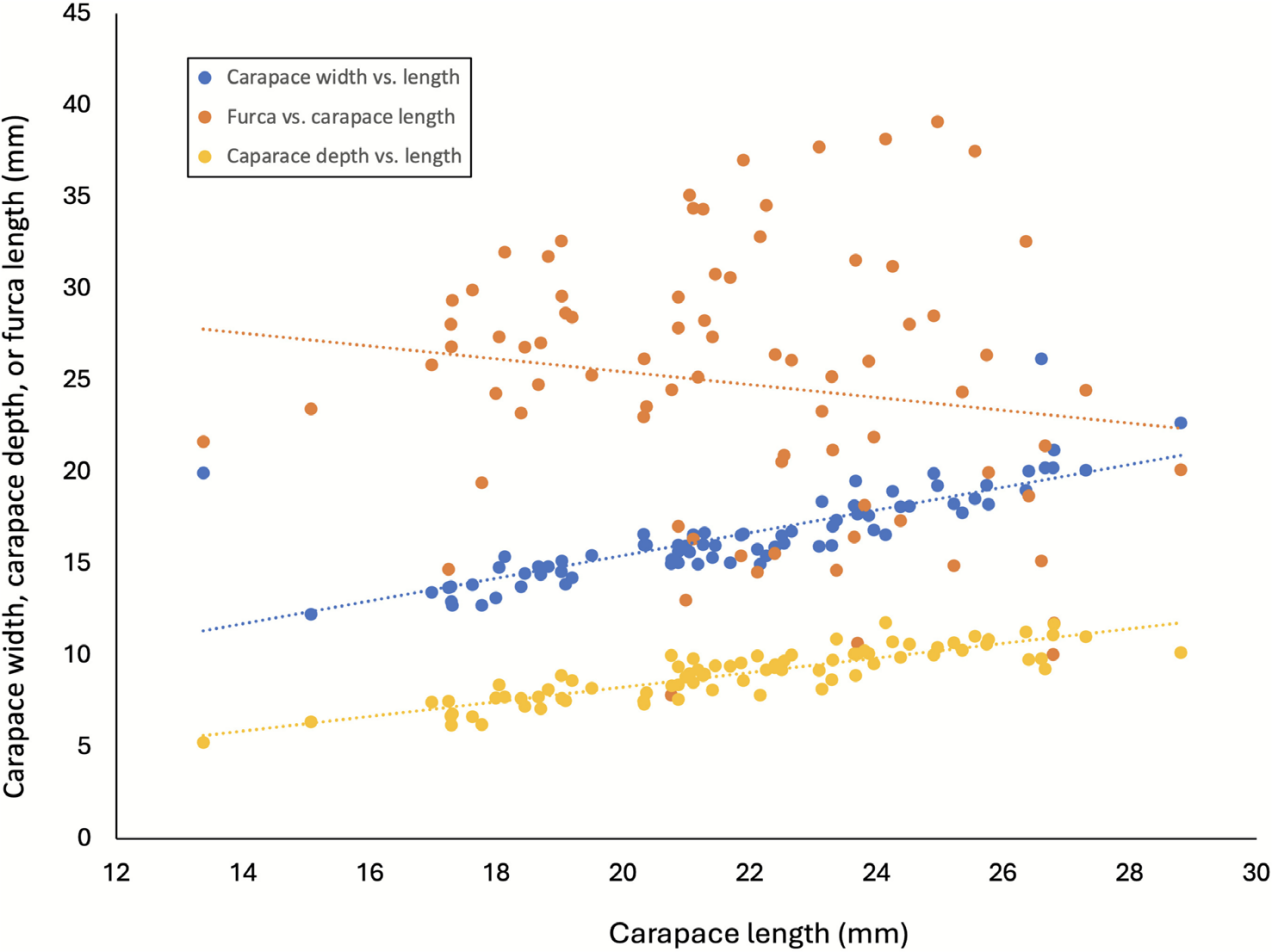
Correlations between the carapace length and width (blue dots), length and depth (yellow dots), and the furca length (orange dots) from 80 specimens. Both the carapace width and carapace depth are strongly correlated with the carapace length (*r* = 0.80 and *r* = 0.87 in order, *p* < 0.0001, df = 78 for both, Pearson correlation) while the furca length shows a slight negative correlation with the carapace length, although the relationship is not significant (*r* = −0.15, *p* > 0.05, df = 78, Pearson correlation).

The carapace length, width, depth, and furca length were measured and their correlations were determined on 80 individuals collected in spring 2022. There was a positive linear correlation between the width and the length of the carapace (*r* = 0.80, *p* < 0.0001, df = 78, Pearson correlation). Similarly, a strong positive correlation was observed between the depth and the length of the carapace (*r* = 0.87, *p* < 0.0001, df = 78, Pearson correlation). On the contrary, a slight negative correlation, although not significant, was observed, between the furca length and the carapace length (*r* = −0.15, *p* > 0.05, df = 78, Pearson correlation) (Figure 6).

Because chemical preservation contracts the lengths of the tails (Longhurst 1955b), the proportion of the carapace length to the length of the exposed abdomen was then measured through lab‐raised specimens. The live specimen reared in the lab (Figure 4) was about 60 days old when the image was captured, where the carapace length to the abdominal length was at the ratio of 1.46. The other specimens (*n* = 4) were about 72 days old when the measurements were recorded and showed an average of 1.36 in the lengths of the carapace to the abdomen.

No males were observed among the 106 collected specimens and eight lab‐raised individuals, with all individuals showing a pair of brood pouches. In the collected specimens, the younger adults showed a higher percentage of being gravid at the time of collection compared to older adults (Table 2). The ages were estimated by the number of days the rain pool was maintained when the collection was made minus one day as the presumable required time for hatching (Table 2).

**TABLE 2:** Percentage of individuals with eggs and their approximate maximum age.

Maximum age (day)	Percent with eggs in brood pouch (sample size)
33	90% (*n* = 24)
40	85% (*n* = 23)
47	33% (*n* = 33)
98	23% (*n* = 22)

### 3.2 Molecular Analysis

**FIGURE 7:**
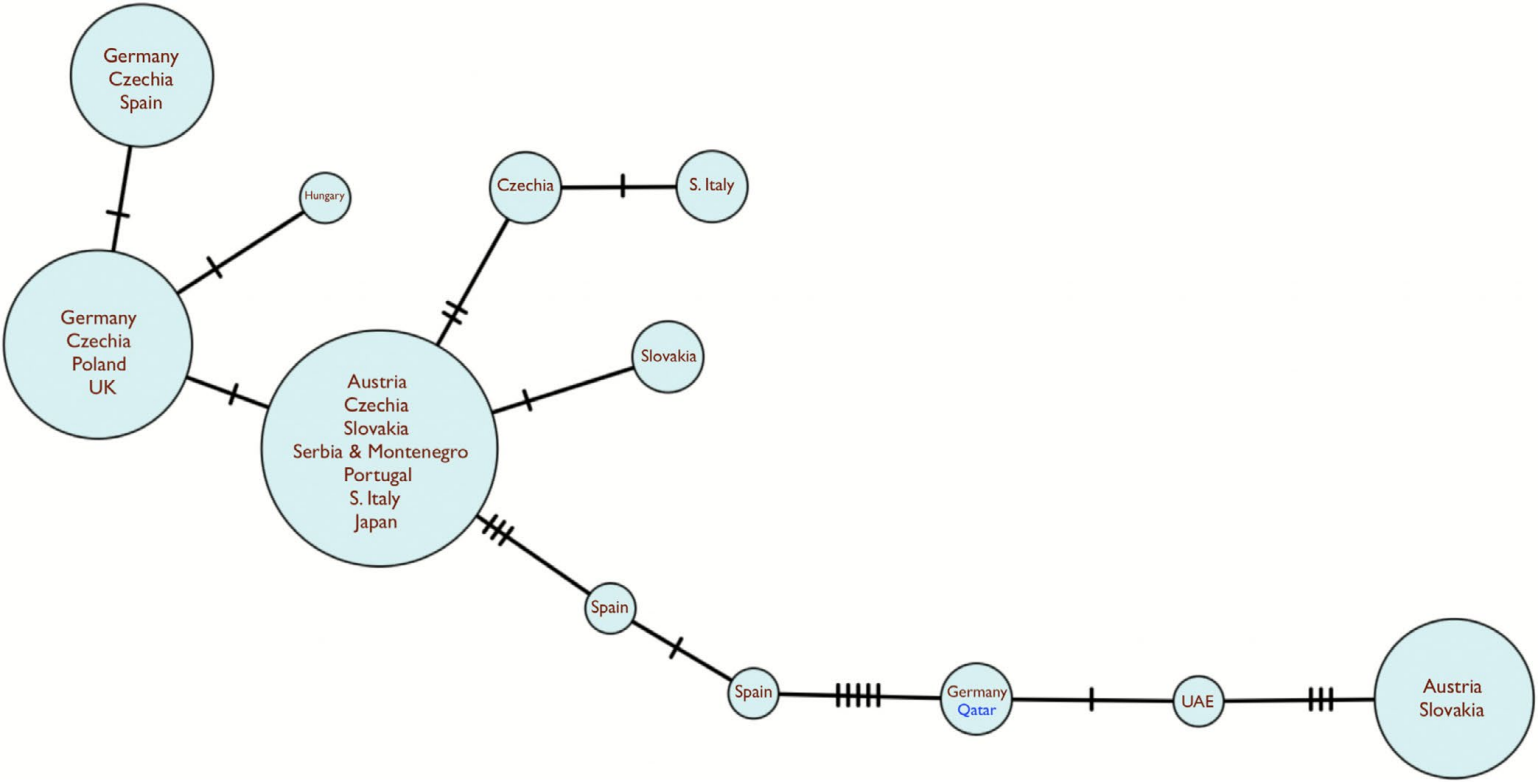
Haplotype network based on COI gene sequences. Only one haplotype was found in 23 samples in the Nu'man population and it is identical to that of the Königswartha population in Germany (Qatari haplotype in blue). That and all other sample sequences were obtained through BLAST searches on NCBI website and those with at least 97% identity were included in this analysis. Each tick mark indicates one nucleotide substitution. Some countries had more non‐identical representative sequences yet were treated as the same by PopART (Leigh and Bryant 2015) because of ambiguous nucleotide bases.

**FIGURE 8:**
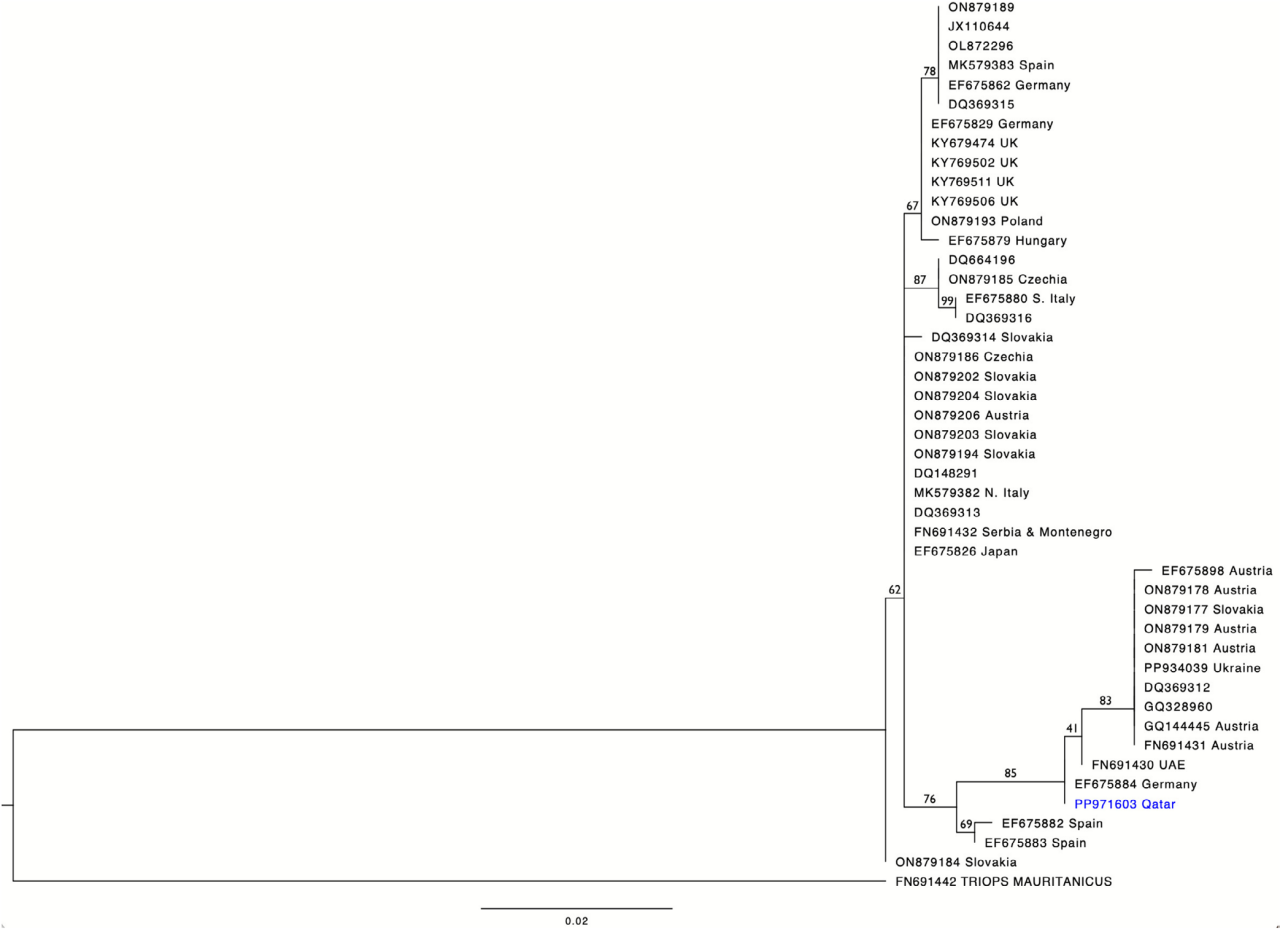
COI sequence‐based maximum likelihood tree depicting the relationship of the Qatari population of *T. cancriformis* with other, similar individuals of presumably the same species. Each sample is shown by its accession number and the values on the branches are bootstrap percentages. Sequence alignments were conducted using Muscle on MEGA v. 11, and the phylogenetic analysis was conducted on raxmlGUI 2.0 using the HKY model and Gamma distribution (median setting) and with 1000 bootstrap replications.

Only one haplotype (Accession no. PP971603) was found in the COI sequence from 23 specimens collected from Rawdat Nu'man. The aligned sequence of 637 base pairs is identical to one haplotype found in Königswartha, Germany (haplotype H13, location code KOE11 of Pond 11) (Zierold et al. 2007), a location near the eastern border of the country neighboring Poland and Czechia. Aside from this German haplotype, all other European samples were at least four nucleotide substitutions away from the haplotype of the Nu'man population (Haplotype network, Figure 7). The only other available sample from the Arabian Peninsula was collected from the United Arab Emirates (Korn et al. 2010), showing one nucleotide substitution from the Qatari samples. In the phylogenetic analysis, the sample from Germany, UAE, and Qatar, along with a few samples from Austria and Slovakia, forms a cluster with strong support of 85% bootstrap value (Figure 8).

**FIGURE 9:**
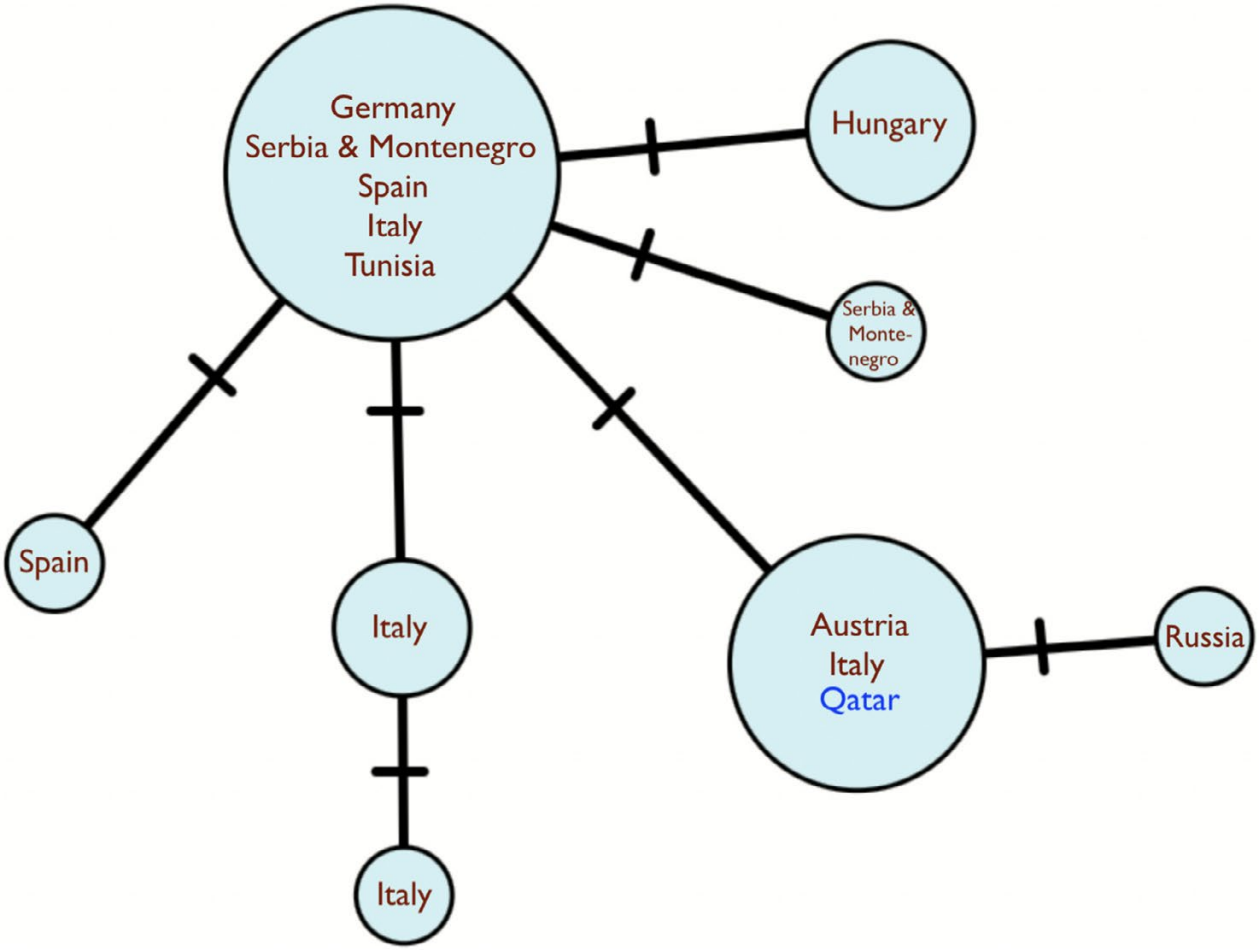
Haplotype network on 16S sequences produced on PopART (Leigh and Bryant 2015) using minimum spanning network setting. Identical sequences from the same country were only represented by one sequence. The node (circle) size is proportional to the number of sequences of the same haplotype. Some circles included sequences from the same country under the minimum span criterion as the uncertain nucleotides, while considered different on NCBI, were considered the same under the PopART setting.

For the 16S sequence, only 14 samples from Nu'man produced resolvable sequences at 521 base pairs, all of which share one single haplotype (GenBank Accession no. PV768972). This haplotype is identical to that in a sample from Austria (Accession no. AM183821, Korn, Marrone, et al. 2006) that overlaps at 515 base pairs and in the representative specimen of Koenemann et al. (2010) (GQ328946) with 521 completely overlapping base pairs yet with no available collection information. An identity of 100% is also assigned to two other, shorter sequences (429 to 492 bases) from Austria and one from Italy, so their full identity with the Qatari *T. cancriformis* haplotype could not be accurately confirmed. In the haplotype network analysis, the 27 sequences were clustered into eight different haplotypes, where ambiguous nucleotides were excluded from being considered for analysis (Figure 9). In this analysis, the Qatari haplotype is clustered with four sequences from Austria and one from Italy. Because of low sequence variation, thus limited phylogenetic information, no further phylogenetic analysis was conducted based on the 16S sequences.

It is worth noting that the Austrian specimen from which the 100% identity 16S (AM183821) was obtained also produced a COI sequence (FN691431) that was included in this study. This COI sequence showed 5 nucleotide substitutions compared to the haplotype found in the Nu'man population and is clustered in the node with other Austrian and Slovakian sequences (Figure 7, rightmost node).

## 4 Discussion

### 4.1 Morphology Supporting *Triops cancriformis simplex*

The members in the Rawdat Nu'man population of *T. cancriformis* bear the morphological characteristics of the *T. c. simplex* subspecies (Longhurst 1955b; Thiéry 1996). The numbers of segments, i.e., thoracic segments and abdominal segments, including apodal segments, are all quite invariable, standing at 33 in 95% (101 of 106) of the sample. The number of apodal segments, at five, is identical in all examined specimens (Table 1). The length, width, and depth of the carapace follow a positive correlation as the size of the individual increases. The abdominal lengths are proportionally short in comparison with the carapace, giving an impression of a large shield with a short “tail.” These observations are within the range of variation in the description of *T. cancriformis* in Longhurst's (1955b) review of the species. In his observations, the abdominal segments were somewhat variable, ranging from 33 to 35, even in the pure line samples reared in the lab. Similarly, the apodal segments might range from five to seven in the pure line *T. cancriformis* in his study, while in the Nu'man population, the number is consistently five in all 106 specimens. The carinal features in the specimens from Rawdat Nu'man are consistent with that of *T. cancriformis simplex*, where this ridge is smooth in its entire length, including the posterior end meeting the sulcus. The smoothness of the posterior part of the carinal shield is also mentioned in *T. c. simplex* from Yemen (Thiéry 1996) and Saudi Arabia (Hassan 2015). The spikes on the sulcus are smaller than those in the subspecies of *T. c. cancriformis*. Also, strong spikes on the lateral sides of the telson in the Nu'man population have been observed in *T. c. simplex* specimens collected from Yemen (Thiéry 1996) and Saudi Arabia (Hassan 2015). All these features are consistent with those in the *T. cancriformis* specimens collected from Rawdat Nu'man.

The strong linear correlations between the carapace length and width indicate the stability in the proportion of these morphological features as the individuals grow in size. Consequently, any of these measurements may be used as a proxy for their size. The furca length, however, is not a reliable prediction of the age or size of the individuals upon reaching sexual maturity, presumably as indicated in this study. It is notable that a slight negative correlation was present in the length of the furca and the carapace (although not statistically significant, Figure 6). This observation may indicate that the length of the furca is maintained and the length no longer increases when the individual reaches a certain level of growth (in this study at about > 30 days) and has reached sexual maturity. This observation is consistent with individuals whose carapace lengths have already reached about 15 mm, upon which the length of furca ceases to increase (fig. 9 of Longhurst 1955b). This is different from the carapace growth, which seems to continue at least during the observation period of this study. The slight reduction of the furca length occurs perhaps through losing a small portion of the delicate tips as a result of wear and tear or as an artifact of handling (personal observation).

### 4.2 Reproduction in the Rawdat Nu'man *T. cancriformis* Population

The reproductive system in notostracans is known to be flexible in which transitions between gonochorism and androdioecy have occurred multiple times in the evolutionary history of the order (Mathers et al. 2013). As a species, *T. cancriformis* shows the widest range of reproductive modes with dioecious (gonochoric), hermaphroditic, and androdioecious populations found throughout its range (Engelmann et al. 1997; Zierold et al. 2007; Mathers et al. 2013). The gonochoric populations, being the more basal reproductive mode of the lineage, are believed to be a relic that survived the postglacial period in the Late Pleistocene in the modern‐day Iberian Peninsula as well as North Africa where populations show high genetic diversity (Zierold et al. 2007). The presence of egg pouches in all (106 plus 8 lab hatched = 114) examined specimens and the absence of male behavior in the field or in lab‐raised specimens suggest that hermaphroditism is the likely reproductive mode in the Rawdat Nu'man population. It has been confirmed that sex determination in *T. cancriformis* follows the ZW chromosomal system where females are the heterogametic sex that determines the sex of the offspring (Mathers et al. 2015). In their study, the males in both the gonochoric (ESP in Spain) and the androdioecious (KOE12 of Königswartha, Germany) populations are homogametic of ZZ chromosomes and the females in the gonochoric populations have the ZW chromosomes. The hermaphrodites in the androdioecious population may be either WW or ZW. Because of the high similarity of the W chromosomes among the gonochoric and androdioecious populations and that the sex determination is by females, it is highly likely that the hermaphrodites evolved from females rather than males (Mathers et al. 2015). In their study, they have also noticed that the Z chromosomes are surprisingly divergent, contrasting with the high similarity in the W chromosomes between the two populations. It is proposed that the variable regions in the Z chromosomes may have been the result of introgression of males after the establishment of the hermaphrodites carrying the W chromosome genetically similar to that of the ESP population (Mathers et al. 2015). It is worth noting that the Nu'man population carries the same COI haplotype type as that of KOE11, which belongs to a separate lineage compared to KOE12. Since the KOE11 population is also androdioecious, multiple male introgression may have occurred in the evolution of the *T. cancriformis* reproductive system. It is yet to be determined if the presumed hermaphrodites in Nu'man are either monogenic WW, amphigenic ZW, or both and if a similar divergent pattern is seen in their Z chromosomes. Future studies may utilize the microsatellite markers already developed for this species to answer the question (Cesari et al. 2004; Zierold et al. 2009).

### 4.3 Comparison With the Taif, KSA Population

The distribution of *T. cancriformis* in Europe is broadly reported, yet its occurrences outside the region are rarely mentioned (but see Suno‐uchi et al. 1997; Golzari et al. 2009; Padhye and Ghate 2016). The population of *T. cancriformis* discovered in Taif, Saudi Arabia, is the closest geographically to the Nu'man population (Hassan 2015). The members in Taif and in Nu'man share a few morphological similarities. One prominent trait is the presence of the second maxillae in both populations. Additionally, the shape of the dorsal organ being round, the absence of supernumeraries on the apodal segments, and the presence of smooth carinae are all shared characteristics between the two populations. However, in the approximately 100 specimens collected from Taif, both males and females existed, and the males reached 37% in one collection (Hassan 2015), while the other was less than 5%. In his study, the number of body segments in females ranged from 32 to 33, while that of the Nu'man population was between 33 and 34. The number of the apodal segments was between five and seven in the Taif population but was invariable at five in all of the Nu'man specimens. Their male members exhibited 32 to 37 body segments, of which the apodal segments ranged from four to seven. Comparing the populations between Taif and Nu'man, the former appears to be more variable, both in morphological traits and in the modes of reproduction. Because of the range of sex ratio, the reproductive mode in the Taif population appears to follow androdioecy. This sex ratio was not observed in a previous study in another Middle Eastern population. In Iran, the population near Urmia Lake in West Azerbaijan Province shows an extremely rare incidence of males, only one in 400 specimens (Golzari et al. 2009). This, again, seems to be another example of androdioecy, with the majority of the members likely to be hermaphrodites. The observation that the entire samples of 114 individuals in the Nu'man samples were all females seems to suggest a hermaphroditic population, yet the possibility of it being androdioecious cannot be ruled out considering the low incidence of males in the Urmia Lake population in Iran (Golzari et al. 2009).

### 4.4 Phylogeny, Phylogeography, and the Origin of the Nu'man Population

*Triops mauritanicus* has been confirmed as the sister taxon of *T. cancriformis* (e.g., Korn et al. 2013). In the phylogenetic analysis using *T. mauritanicus* as the outgroup with the available COI sequences on GenBank, the population in Nu'man is shown to be closest to those found in eastern Germany and other central European countries (Figure 8). The results in the 16S haplotype network are consistent with this observation, despite this marker showing lower resolution in the lineages. The three members in Königswartha that showed the identical COI sequence with the samples in Nu'man were identified as *T. c. cancriformis* (Zierold et al. 2007). Additionally, the specimen collected in UAE is also identified as *T. c. cancriformis* (Korn et al. 2010). Yet the morphology of all specimens in Nu'man is overall consistent with that of *T. c. simplex* (Longhurst 1955b). Further, in the 16S sequence analysis, the identical sequence with the Nu'man haplotype was also identified as *T. c. cancriformis* (Korn, Marrone, et al. 2006). It has been suggested that the morphologically described subspecies of *T. c. simplex* and *T. c. cancriformis* are not supported as distinct monophyletic lineages based on nucleotide‐based phylogenies where each nominal subspecies is paraphyletic (Korn, Marrone, et al. 2006; Zierold et al. 2007). Given the inconsistencies between the support of morphology‐based subspecies and the lack of distinct lineage using molecular data, there is a need to reassess the subspecies status of *T. cancriformis* where the two subspecies may be considered as one that shows a range of morphological variation, mostly in the spines on the carina and the telson.

The evolution of the hermaphroditic and androdioecious modes has facilitated post‐glacial range expansion and colonization northward and eastward to the rest of Europe (Zierold et al. 2007). Consistent with this hypothesis, the populations outside of Iberia exhibit low gene diversity, indicating a more recent evolution consequent to the founding events (Zierold et al. 2009). Further to the observation of long‐distance colonization, the members in the Nu'man population exhibit only one haplotype in both the COI and 16S markers. All examined members showed female morphological traits and no male reproductive behavior was observed either in the wild or in the lab‐raised individuals, which makes it highly likely a hermaphroditic population. Since only one population of *T. cancriformis* is found in Qatar, contrasting a wide distribution of *T. granarius* (Chen et al. 2023), the colonization of Nu'man is consistent with a much more recent event, likely much later than the late Pleistocene expansion of the central European populations. Further, the founders of the Nu'man population are likely from central European populations, specifically in the Königswartha area of east Germany as evidenced by the COI sequence data. As to the 16S data, although in the Nu'man population the sequence is identical to that of an Austrian specimen (except it being a shorter sequence), this same specimen showed a higher divergence in its COI sequence at five nucleotide positions different from that of the Nu'man individuals. Further, 16S sequence being a marker of slightly lower mutation rate in arthropods including in *Triops* (e.g., Papadopoulou et al. 2010; Guy‐Haim et al. 2018; Chen et al. 2023), and in the sequences available on GenBank as represented in haplotype network study (Figure 9), more populations may share the same haplotype. So the 16S data support the close relatedness yet COI characters may point to a more definitive origin of the Nu'man population. Although these data show support of the close relationships, *T. cancriformis* data outside Europe are scarce. Additional studies, e.g., adding the ATPase gene sequence to the analysis of the Nu'man population and including the 16S information to the Königswartha population may help further confirm the origin of the Qatari population.

In the COI‐based phylogeny, populations located within the same countries or in close geographic proximity may cluster with populations occurring in more distant locations (Figure 8). For example, a Spanish sample (MK579383) is a close relative of a German specimen (EF675862), while another two Spanish individuals (EF675882 and EF675883) are grouped in a separate cluster. Similarly, one German sample (EF675884), being the closest relative of the Qatari population, is more distant from the other two German specimens in this study. This phylogeographic pattern is likely attributed to the ease of dispersal of *Triops* and other aquatic invertebrates, whose diapausing cysts ingested by avian predators promote long‐range migration and colonization (e.g., Santamaría et al. 2023). As gonochorism is a more ancestral reproductive mode, it likely evolved in Pleistocene Iberia. The hermaphroditic *T. cancriformis* population found more recently in Portugal indicates possible recolonization consequent to avian vectors carrying *T. cancriformis* cysts from southern France or northern Italy (Machado et al. 2017). Their phylogeography reveals a complex pattern of lineages and locations. Previously, it has been suggested that the advantage of androdioecy and hermaphroditism in colonizing new territories in high latitudes is present in *T. cancriformis* (e.g., Mathers et al. 2013). As the information on outlying populations, including that in Qatar, has expanded, the advantage of androdioecy/hermaphroditism in dispersal should not be viewed only as a function of high latitudes. It should instead be understood as the superiority of these reproductive modes, hermaphroditism in particular, in supporting long‐range expansion of the species distribution from their original populations (see Section 4.5).

### 4.5 Long Distance Dispersal

Long‐distance bird migration is responsible for the colonization and expansion of the distribution of plants, invertebrates, and microbes globally (Viana et al. 2016). Dispersal of aquatic invertebrates to new aquatic environments can be conducted directly by waterbirds as compared to wind or precipitation since the former readily connect the disjointed water bodies together (Green and Figuerola 2005, Figuerola et al. 2005). The mechanism of range expansion in notostracans including *Triops cancriformis* through avian vectors has been explored in many studies (e.g., Longhurst 1955a; Golzari et al. 2009; Adams et al. 2014; Muñoz et al. 2013; Machado et al. 2017). Hermaphroditism and androdioecy in *T. cancriformis* further facilitate the colonization through avian dispersal. This is evident in the observation that only females are found in outlying populations distant from Iberia or from the central mass in America in the case of *T. longicaudatus* (Longhurst 1955a). Although short‐range dispersal of *T. cancriformis* may be accomplished with wind actions (Adams et al. 2014), long‐distance dispersal often requires avian vectors. Located along the Eurasia and East Africa flyways, the Arabian Peninsula often serves as a stopover point during the avian biannual migration with some species even staying overwinter (e.g., Cramer et al. 2025). While ingesting invertebrates for food, these bird species may carry the propagules such as diapausing cysts internally or externally and deposit them along the route. A recent study has indicated that dispersal through avian vectors may reach up to hundreds of kilometers (Santamaría et al. 2023). Despite this long distance, the colonization of the Qatari population is unlikely to be achieved without multiple layovers given the distance of more than 4000 km between Königswartha and Qatar, assuming the former were the location of the founding population to Nu'man. As it is shown, most of the available genetic information (i.e., COI, 16S, 28S, 12S) is limited to European populations. The closest geographic location to the Middle East with available sequence information is in Cyprus (Tziortzis et al. 2014, but see FAFLP data in Sorek et al. 2016) with scant molecular data from other regions. Beyond Europe, the collection data are scarce, let alone genetic information. This knowledge gap in the *T. cancriformis* populations outside of Europe impedes the understanding of its dispersal pattern, even if the presence of the species has been reported in a few Middle Eastern countries (Thiéry 1996; Golzari et al. 2009; Hassan 2015; Rogers et al. 2022). This study marks the first attempt to fill the gap of molecular information in the biogeography of *T. cancriformis* with the focus in the Middle East. Future studies should expand molecular analysis of populations in the Arabian Peninsula, other regions in the Middle East, Eurasia, and elsewhere, especially on the samples where morphological studies have already been completed.

### 4.6 Co‐Occurrence of *Triops cancriformis* and *Triops granarius*

It is worth noting that in the Nu'man Rawdat basin another species of large branchiopod has also been identified. This species, *Triops granarius* s.l., shows a much broader distribution in Qatar, found in many rain pools north of the Dukhan Speedway that traverses the peninsula from Doha in the east to Dukhan on the west coast (Chen et al. 2023). Aside from being more broadly distributed in Qatar compared to *T. cancriformis* , *T. granarius* shows a much shorter life span (up to a month), is gonochoric, and appears to be quite divergent genetically as compared even to the most closely related populations in East Asia (Chen et al. 2023). This suggests that *T. granarius* is a much longer‐term resident in Qatar compared to the newcomer of *T. cancriformis* . The co‐occurrence of the two species in a habitat with a limited time span is worthy of a new ecological study.

## 5 Conclusion

In Qatar, five confirmed species of large branchiopods have been reported, including two species of anostracans, two species of spinicaudatans, and one species of notostracan (Rogers et al. 2022). This study confirms the second species of notostracan, *T. cancriformis* , in the eastern Arabian Peninsula based on both morphological and molecular characteristics. Being all hermaphrodites in this population is consistent with the feasibility of long‐distance colonization of new territory facilitated by possibly avian seasonal migration through Eurasia–East Africa flyways. The high uniformity among members of the Nu'man population, both in morphology and molecular characteristics, and the limited distribution in Qatar all suggest it results from recent colonization.

## Author Contributions

**Kuei‐Chiu Chen:** conceptualization (lead), data curation (lead), formal analysis (lead), funding acquisition (lead), investigation (lead), methodology (lead), project administration (lead), resources (lead), supervision (lead), validation (lead), writing – original draft (lead), writing – review and editing (lead). **Ramaswamy Narayanaswamy:** data curation (supporting), formal analysis (supporting), investigation (supporting), methodology (supporting), validation (supporting), writing – original draft (supporting), writing – review and editing (supporting).

## Funding

This work was supported by Weill Cornell Medicine ‐ Qatar, Faculty Discretionary Fund. Open access funding for this article was provided by the Premedical Division of Weill Cornell Medicine ‐ Qatar.

## Disclosure

The collections were approved under the Qatar Ministry of Municipality and Environment (MME) IACUC Registration No.: MoPH‐MME‐003, and Assurance No.: IACUC‐A‐004.

## Conflicts of Interest

The authors declare no conflicts of interest.

## Data Availability

The data supporting the findings of this study is available through this dataset https://doi.org/10.5061/dryad.0zpc8679n.
